# A Computational Model for Evaluating Transient Auditory Storage of Acoustic Features in Normal Listeners

**DOI:** 10.3390/s22135033

**Published:** 2022-07-04

**Authors:** Nannan Zong, Meihong Wu

**Affiliations:** School of Informatics, Xiamen University, Xiamen 361005, China; 31520190154747@stu.xmu.edu.cn

**Keywords:** sensitivity, acoustic features, instantaneous change in correlation, interaural delay, auditory perception, computational model, interaural coherence

## Abstract

Humans are able to detect an instantaneous change in correlation, demonstrating an ability to temporally process extremely rapid changes in interaural configurations. This temporal dynamic is correlated with human listeners’ ability to store acoustic features in a transient auditory manner. The present study investigated whether the ability of transient auditory storage of acoustic features was affected by the interaural delay, which was assessed by measuring the sensitivity for detecting the instantaneous change in correlation for both wideband and narrowband correlated noise with various interaural delays. Furthermore, whether an instantaneous change in correlation between correlated interaural narrowband or wideband noise was detectable when introducing the longest interaural delay was investigated. Then, an auditory computational description model was applied to explore the relationship between wideband and narrowband simulation noise with various center frequencies in the auditory processes of lower-level transient memory of acoustic features. The computing results indicate that low-frequency information dominated perception and was more distinguishable in length than the high-frequency components, and the longest interaural delay for narrowband noise signals was highly correlated with that for wideband noise signals in the dynamic process of auditory perception.

## 1. Introduction

The concept of auditory perception seems so simple. People can easily hear the music of the neighborhood piano, detect the sound of nearby passing cars, and perceive the sound of waves in the distance [1]. On the surface, auditory experience is simple and effective, but there are still many difficulties and questions that have not been adequately addressed [2]. In particular, the information that reaches our hearing is usually incomplete and fuzzy, distributed in space and time, and is not sorted neatly according to its source [3]. Obviously, in order to support effective interaction with the world, human listeners need to extract a lot of information about the world from sound [4].

Notably, the auditory system of humans confronts the natural pressure to manage sound wave reflections in ordinary living conditions on a daily basis. In a noisy, reverberant environment, listeners receive not only sound waves directly originating from sound sources but also reflections of the sound sources [5]. That is, there are not only direct sound waves from the sound source but also reflected sound waves from the surface of surrounding objects, and the reflected sound always lags behind the direct sound in reaching the human ear [6]. In order to segregate the target signal perceptually from other interference stimuli in the reverberation environment, the auditory system must simultaneously distinguish the sound wave directly from the signal source with the reflection of the signal source in addition to the sound wave from the interference source with the reflection of the interference source [7]. Otherwise, the auditory scene will become chaotic. Thus, under adverse hearing conditions, the high perception fusion tendency of acoustic signals is conducive to speech recognition.

Since auditory information is processed in a time sequential mode, the auditory transient “memory” and sequential auditory information readout from the auditory transient storage are very important for organizing sound stimuli components into auditory image units [5]. Therefore, the auditory system must make use of the acoustic features of direct sound retained in the initial auditory “memory” to carry out correlation “calculation” with the acoustic features of the reflected sound, and then conduct perceptual integration of the direct and reflected sound information to form the perception of a single auditory event [7]. Thus, the transient “memory” of the acoustic features is crucial for accurate localization of sound incidence in an everyday environment [5,7].

It has been well confirmed that due to the ability to transiently store raw acoustic features of the leading wave, the auditory system of human listeners can calculate the similarity between the leading and lagging sound waves and represent the calculation result at the perceptual level [8,9]. Previous studies have discovered that the ability to transiently store acoustic features contributes to the perceptual integration of the direct sound wave and its time-delayed reflections [10,11,12]. It has also been found that the acoustic features of waves coming directly from the sound source must be transiently held for a short period to achieve integration of the direct acoustic wave with its time-delayed reflection [13,14]. The remarkable ability to transiently store acoustic features enables the auditory system to recognize whether acoustic features of one wave are highly correlated with that of another wave. This is beneficial for speech recognition in noisy environments. There is evidence showing that speech performance under a noisy environment was strongly correlated with the ability of temporal storage capacity of acoustic features [10,11]. The ability to transiently store acoustic features is critical for perceptual segregation of target speech and an informational speech masker in a simulated noisy and reverberant environment [11]. This interaural correlation processing ability plays an important role in improving speech recognition by inducing a perceived spatial separation between target source and the other masking source. Thus, detecting a change in correlation is an essential component of auditory scene analysis. For correlation comparison, transient auditory storage of acoustic features is necessary [14]. Therefore, the ability to temporally store acoustical features is critical for later speech perception in noisy environments [11].

There has been increasing interest in discussing how to assess this remarkable ability to transiently store acoustic features and elucidating methods to provide a logical description of the calculation process of interaural correlated acoustical information in auditory scene analysis. Therefore, this study aims to explore methods to evaluate the ability of transient “memory” of acoustic features qualitatively.

There are many ways to represent auditory perception information, such as classical psychophysical methods for estimation (e.g., estimation of difference threshold [15,16,17] or only significant difference [8]), non-parametric Bayesian clustering algorithms for representation of auditory cues [18], and deep learning methods [19,20] for single and multiple sound source localization. These studies indicate that the minimal difference between two stimuli leads to a mathematical approach to relating to the external physical world based on experimental data and the internal psychic response. Delving further, researchers have found that the processing of interaural correlation could be investigated by measuring the sensitivity to an instantaneous change in interaural correlated steady-state noise [8]. This method, in which an instantaneous change in interaural correlation is created by placing an uncorrelated noise burst between two noise stimuli with identical waveforms, has been widely used for binaural gap detection. In other words, incoherence is introduced in this kind of method; for example, when an unrelated noise is added to the acoustic signal appearing in one of the ears. It can be seen that this instantaneous change sensitivity helps to assess the influence of the listener on auditory perception.

It has been well documented that humans can detect the binaural gap in interaural correlated steady-state noise when the interaural correlated noise arrives simultaneously [8,12,21]. Interestingly, when an interaural time difference [22] (i.e., interaural delay) was introduced, the binaural gap in interaural correlated steady-state noise was still detectable [23,24]. Since the acoustic signals in the left and right ears differ in some way, such as a simple time delay, the interaural incoherence occurs when measuring the similarity between the binaural sound inputs. The preservation of the sensitivity to the binaural gap even when an interaural delay was introduced indicated that acoustic temporal fine structure features of noise were maintained for the duration of the interaural interval [11,25,26] and allowed similarity computation between the binaural sound inputs. Thus, measuring the impact of interaural delay when the binaural gap is detected [27,28,29] can provide a way of investigating the transient memory of acoustic features.

Since a binaural gap is detected when the noise sections flanking the binaural “gap” are sufficiently large [27,28], the shortest detectable binaural gap could be one measure of the temporal response of the binaural system to instantaneous changes in interaural correlation. In the meantime, the transient memory of acoustic features of the noise signal entering the leading ear continues to attenuate and the interaural correlation of flanking the break-in correlation in the central representation gradually decreases. If the interaural integration of acoustic features (i.e., transient memory of acoustic features) is degraded by introducing an interaural delay, then the detectability of the binaural gap should be reduced, resulting in decreased sensitivity for binaural gap detection. Thus, the dynamic characteristic of transient memory of acoustic features can be reflected by the variability in the duration threshold for the binaural gap, detectable because the interaural delay is varied. However, to the best of our knowledge, whether the sensitivity to the binaural gap affects the interaural delay when the binaural gap is detected has not been well discussed in the previous literature.

In the present study, whether the ability to transiently store acoustic features is affected by the interaural delay was assessed by measuring the sensitivity for detecting the binaural gap at various interaural delays. Moreover, the present study also contributes to a discussion about whether the sensitivity to the binaural gap is related to transient memory of acoustic features. It is well known that the auditory system is extraordinarily capable of processing acoustic features [21]. There is evidence in a previous study that the jitter of interaural time differences in uncorrelated signals was determined by the fine structure acoustic feature and envelope modulations inherent to the stimulus and the bandwidth of the stimulus, and the absolute bandwidth of low-frequency noise bands is lower than that of high-frequency noise bands when the relative bandwidth is fixed [11]. However, to our knowledge, whether the change in absolute bandwidth affects the ability to transiently store acoustic features has not been well documented. In addition, whether this binaural ability is maintained when there is a break in a correlation between the interaural correlated narrowband and wideband noise, or whether the ability to temporally store high-frequency acoustic features is associated with the ability to transiently store low-frequency acoustic features, is still unclear.

Research has shown that narrowband noise might have a much higher perceptual fusion tendency compared to wideband noise [30]. Although wideband noise is closer to natural noise, regular narrowband noise more easily attracts attention. Moreover, compared with narrowband noise, wideband noise has a greater impact on the hearing and on the intelligibility of speech. Especially under a low signal-to-noise ratio, wideband noise will cause serious interference and damage to speech perception in noise. Thus, it is also important to investigate the influence of the components (i.e., which attribute) of wideband noise or narrowband noise on the ability of auditory transient memory of acoustic features of a random noise in the sound source. Whether maintaining information of auditory stimuli is dependent on interaural time delay or the frequency component also needs clarification.

Therefore, this study examined the detectability of a binaural gap between interaural correlated narrowband or wideband noise when the longest interaural delay was introduced and measured the ability to temporally store acoustic details associated with the relationship between the transient storage of fine acoustic features of interaural wideband noise and narrowband correlated noise at different center frequencies. Moreover, the present study further builds a paradigm of a computational description model to explore the relationship between wideband and narrowband correlated noise with various center frequencies on the binaural ability to dynamically process acoustic details. Modeling the important functions of human hearing can provide an important opening for the development of a class of promising techniques. The main purpose of this modelling is to describe the evidence for the nature of human auditory organization and to explore the computational models which have been motivated by such evidence. Traditionally, behavioral tests are used to determine the sensitivity of the binaural gap detection in interaural correlation change. Although the behavioral temporal processing test is well established, the underlying mechanisms are not completely understood. These psychoacoustic characteristics of temporal storage capacity of acoustic features are helpful for understanding acoustic performance in reverberant environments. In addition, behavioral tests rely on the subjective reports of participants. An objective approach is useful for testing the sensitivity to the binaural gap detection in interaural correlation change. Hence, an intuitive approach is needed to evaluate the listener’s ability to temporally store acoustic features. The computational models will be much more amenable to objective evaluation, and such evaluations will contribute to a more effective synergy between hearing research and speech processing research.

## 2. Materials and Methods

### 2.1. Participants

A total of 14 young students (9 females and 5 males, mean age = 22.786 years, from 20 to 25 years old) participated in this study. The participants all had normal and bilaterally balanced pure-tone hearing thresholds. All the participants gave their written informed consent to participate in the study. The experimental procedures of the present study involving human subjects were approved by the ethics review committee in Xiamen University.

### 2.2. Apparatus and Materials

The participants were tested individually in a quiet room. Participants listened to stimuli sequences via headphones (HD 265 linear, Sennheiser, Germany). Stimuli were generated with MATLAB (R2019b, the MathWorks Inc., Natick, MA, USA) software running on a PC (processor Intel(R) Core(TM) i7-8700 CPU @ 3.20ghz 3.19ghz, RAM 8 GB) with Windows 10 system. Gaussian wideband noise signal bursts were generated at a sampling rate of 48 kHz for the wideband noise stimulation condition. In the narrowband noise stimulation condition, signal stimuli had a fixed bandwidth of 1/3 octave and center frequencies of 0.2, 0.4, 0.8, 1.6, or 3.2 kHz. Each noise signal with a duration of 2000 ms was created, and presented over two headphones to listeners. The sound level was set at 60 dB sound pressure level, which was calibrated by the Larson Davis Audiometer Calibration and Electroacoustic Testing System (AUDit and System 824, Larson Davis, Depew, NY, USA) with “A” weighting.

### 2.3. Design and Procedure

**Figure 1:**
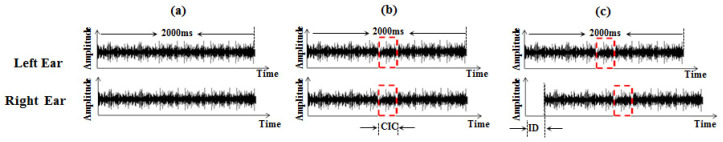
Illustration of the concept of CIC and ID in an example of steady-state noise. (**a**) Shows the two asynchronous but interaural correlated steady-state noise signals. (**b**) Shows an instantaneous change in correlation (CIC) inserted into the two asynchronous but interaural correlated steady-state noise signals. (**c**) Shows an interaural delay that was introduced on the basis of (**b**) for the measurement of the ability to transiently store acoustic features.

Each trial in a testing block included two 2000 ms intervals of correlated noise (Figure 1a). In one interval, the right-headphone noise was the same as that of the left-headphone noise. In the other interval, the right-headphone noise was also identical to the left-headphone noise, except that the temporal middle of the 2000 ms noise was substituted with an interaural uncorrelated noise segment, in which this substituted noise segment introduced an instantaneous change in correlation (CIC) (Figure 1b) [10]. An interaural delay (ID) was introduced in the form of an interaural delay between the two intervals, which determined the interaural coherence of the interaural correlated noise (Figure 1c).

Participants performed an adaptive two-interval, two-alternative, forced-choice task track with visual indications of the listening intervals. The task of the participants was to identify which of the two intervals contained CIC (the binaural “gap”) immediately after the stimuli ended. During the experiment of this study, the duration of the CIC was fixed at 2000 ms according to the experiment explored in Huang et al., (2009) [10].

In each test, the probability that CIC was assigned to one of the two intervals was equal. The time interval separating the two intervals was set as 1000 ms. For each interval, the noise signals from the left earphone and those from the right earphone started simultaneously, and the duration of ID was systematically adjusted. New generated noise signals were used for each trial. The order of noise signal types was counterbalanced across participants using a Latin square order. The participant started a trial by pressing a command button on the keypad. The longest ID for CIC detection was measured and the duration length of ID for CIC detection was adjusted in a 3-down, 1-up procedure [31].

Screening procedures required the listener to identify each of the two 2000 ms intervals and they were asked to respond as soon as possible after the stimuli ended. The order name for each interval was presented on a computer monitor immediately prior to and during each trial.

The feedback on participants’ accuracy was displayed on a computer monitor immediately after each trial and responses were collected through a standard computer keyboard.

## 3. Computational Model and Logical Paradigm

A computational description model for initializing the ability to transiently store the acoustic details is described in the following.

(1) Let the 2000 ms interval of correlated noise signals be denoted as *h*_1_(*t*), and the independent 200 ms noise segment that was interaural uncorrelated with *h*_1_(*t*) be denoted as *h*_2_(*t*). The noise signals presented to the left ear and right ear are indicated as *h_L_*(*t*) and *h_R_*(*t*), respectively, and were constructed by mixing *h*_1_(*t*) and *h*_2_(*t*) according to the following equations:

(1) $$\mathrm{Interval}\;1:\;h_{L}\left(t\right)=h_{1}\left(t\right);h_{R}\left(t\right)=h_{1}\left(t\right).$$

(2) $$\mathrm{Interval}\;2:\;h_{L}\left(t\right)=h_{1}\left(t\right);h_{R}\left(t\right)=\alpha h_{1}\left(t\right)+h_{2}\left(t\right)+\left(1-\alpha -\frac{\left|h_{2}\left(t\right)\right|}{\left|h_{1}\left(t\right)\right|}\right)h_{1}\left(t\right).$$

(2) Using the three-up, one-down procedure to obtain the longest ID for each session.

After three consecutive correct identification intervals containing CIC, the duration of ID was increased, and following one false identification, the duration of ID was decreased. According to this principle, let *p* be the probability of a positive response during a test, then the probability of obtaining an increased response sequence is denoted as $p^{3}$, and the probability of obtaining a decreased response sequence is indicated as $p^{2}\left(1-p\right)+p\left(1-p\right)+\left(1-p\right)$.

The procedure that systematically manipulates the interaural interval and computes the mean value over several sessions of the ID for each participant was developed to realize the function Φ, which was described in Levitt (1971) [31]. Let ${\bar{t}}_{IAI}$ denote the mean value over several sessions of the ID measures for each participant, which was calculated as follows:

(3) $${\bar{t}}_{IAI}=\Phi \left(H\left(t\right)\right)$$

(3) Correlation coefficients [32] between the longest ID for wideband noise and those for various center frequencies of narrowband noise.

There are two stimulation conditions in this study: in narrowband noise stimulation conditions, for participant *i*(i=1,2,⋯,n), the longest ID for which each participant can detect 2000 ms CIC in narrowband noise with different center frequencies is indicated as $\bar{t_{i}}$(i=1,2,⋯,n), and $\bar{t_{ij}}$ denotes the longest ID at which the CIC could be detected in the narrowband noise with the *j*th (*j* = 1,2,⋯,*m*) kind of center frequency for participant *i*. In wideband noise stimulation conditions, $T_{i}$(i=1,2,⋯,n) denotes the longest ID at which the CIC in the wideband noise could be detected for participant *i*.

Let ${\bar{\omega }}_{j}$ denote the weight degree of contribution that the *j*th (*j* = 1,2,⋯,*m*) kind of center frequency component makes to the auditory storage of the temporal storage of wideband noise features. Then, the calculation between the variability of the longest ID for wideband noise across participants and the longest ID for narrowband noise is indicated as follows:

(4) $$\sum_{j=1}^{m}\bar{t_{ij}}\times {\bar{\omega }}_{j}=T_{i}\left(i=1\cdots n\right)\;$$

where $\sum_{j=1}^{m}{\bar{\omega }}_{j}=1$; ${\bar{\omega }}_{j}\gg 0$.

(4) Find the best fit of ${\bar{\omega }}_{j}\;$ from the participants’ data. This could be solved by a linear least-squares problem with linear constraints [33]. If given the data matrix $t_{ij}$, ${\bar{\omega }}_{j}$, then we could deduce the variability of the longest ID for wideband noise only from the narrowband noise across participants, and the mean longest ID at which the CIC in the wideband noise could be detected in wideband noise stimulation conditions. Let ${Τ}^{\prime }$ denote the mean longest ID at which the CIC in the wideband noise could be detected in wideband noise stimulation conditions, which was calculated as a function of each center frequency $\bar{t_{ij}}$ as follows:
  (5) $$T^{\prime }=\frac{\sum_{i=1}^{n}T_{i}}{n},T^{\prime }=\frac{\sum_{i=1}^{n}\sum_{j=1}^{m}\bar{t_{ij}}\times {\bar{\omega }}_{j}}{n}$$

(5) As *T*_i_ denotes the longest ID for participant *i*, let $T_{\delta }$ denote the ID at which the CIC in the wideband noise could be detected in wideband noise stimulation conditions, and let $\rho \left(T_{\delta }\right)$ denote the interaural correlation coefficient between the signals at the two ears, and let ϑ denote the maximum correlation coefficient. Thus, the maximum correlation coefficient ϑ was calculated as follows:
  (6) $$\vartheta =\underset{T_{\delta }}{\max}\left(\rho \left(T_{\delta }\right)\right)=\underset{T_{\delta }}{\max}\left(\frac{\int_{-\infty }^{\infty }h_{L}\left(t\right)h_{R}\left(t-T_{\delta }\right)dt}{\sqrt{\int_{-\infty }^{\infty }h_{L}{\left(t\right)}^{2}dt}\sqrt{\int_{-\infty }^{\infty }h_{R}{\left(t\right)}^{2}dt}}\right)$$

Let ${Τ}_{\mu_{i}}$ denote the duration threshold for binaural gap detected of participant *i*; thus, the duration threshold for the binaural gap detected at various ${Τ}_{\delta }$ can be calculated as follows:

(7) $$T_{\mu_{i}}=T_{\lambda }+T_{\beta }\times \rho \left(T_{\delta }\right)+T_{\gamma }{}_{{}_{i}}+N\left(0,\sigma \right)$$

Thus, threshold ${Τ}_{\mu_{i}}$ includes a fixed intercept ${Τ}_{\lambda }$ and an interaction term ${Τ}_{\beta }$. The decision variable β in multiple observation tasks is a weighted sum of the observation $\beta =\sum \xi_{k}x_{k}$, where $x_{k}$(*k* = 1, 2, ..., *z*) denotes the *k*th observation and $\xi_{k}$ denotes the associated decision weight. Thus, the set of weights could represent a listener’s decision strategy, and decision weights were estimated for each participant across conditions. Moreover, the differences in individuals were modeled by including random intercept $\gamma_{i}$, and *N*(0, *σ*) denotes the measurement error, which is modeled as a normal distribution with standard deviation σ and zero mean.

In the present study, auditory trials recorded the quantitative measures of detected intervals and characterized the longest detectable instantaneous change in correlation for both wideband and narrowband correlated noise in the presence of the longest interaural delay.

## 4. Results

**Figure 2:**
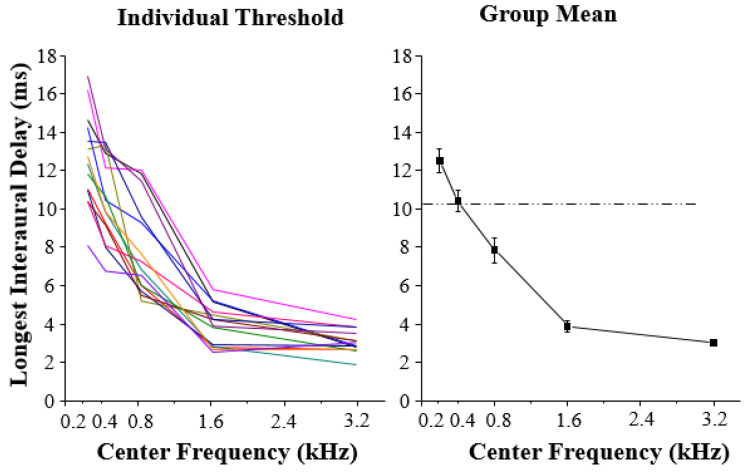
Individual thresholds (left panel) and group mean threshold (right panel) of the longest ID at which the CIC in the narrowband simulation noise could be detected as a function of the center frequency. The dash-dotted line represents the longest ID when the noise type was simulation wideband noise. The different color line represents the data of each individual.

A one-way within-participant analysis of variance showed that there was a significant difference in the main effect of noise type (F_5, 65_ = 107.770, *p* < 0.001). Post hoc analyses with the significance level set at 0.00333 indicated that there was a significant difference between the longest ID for wideband simulation noise and for narrowband simulation noise with center frequencies of 0.2, 0.4, 0.8, 1.6, and 3.2 kHz (all *p* ≤ 0.002). As shown in Figure 2, the longest ID for center frequencies of 0.2, 0.4, or 0.8 kHz was significantly longer than that for narrowband simulation noise with center frequencies of 1.6 or 3.2 kHz. The results indicate that the performance of participants is generally better when the type of center frequency of narrowband simulation noise is in a low stage than that when the center frequency is in a high stage. This result also suggests that the duration time storage of low-frequency acoustic features lasts longer than that of high-frequency acoustic features.

To further investigate the relationship between the contribution that low-frequency components and high-frequency components make to the auditory storage of acoustical details in the dynamic process of auditory perception, a mathematical linear model with statistical computer packages in Matlab was used to generate the best fitting curve for participants’ data. The linear model was adopted because it involves the simplest and seemingly most restrictive statistical properties: independence, normality, constancy of variance, and linearity. The group-mean longest ID at which the CIC could be detected in the narrowband noise as a function of center frequencies was examined. The best fitting psychometric function was used for describing the relationship between the longest ID and the center frequencies, and the obtained value of quantitative goodness-of-fit of models to data was 0.9987. The simulation model can reveal that in the auditory process of low-level transient storage of acoustic details, low-frequency components and high-frequency components contribute differently to the auditory storage of broadband details.

**Figure 3:**
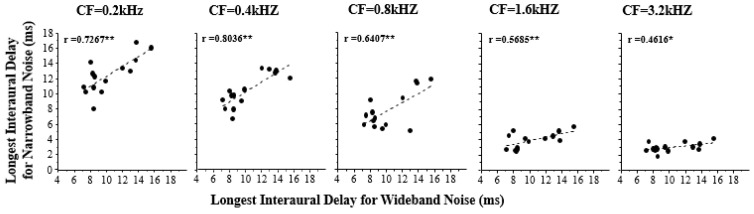
The longest ID at which a temporal break in correlation could be detected for each of the five center frequencies (CFs) of narrowband simulation noise as a function of that for wideband simulation noise. *, indicates significant at the level of 0.05; **, indicates significance at the level of 0.01.

Figure 3 shows the correlation between the longest ID at which a CIC could be detected for various center frequencies of narrowband simulation noise and that for wideband simulation noise. The results suggest that the temporal storing of low-frequency acoustic features lasted longer than that of high-frequency acoustic features. Furthermore, the longest ID for the wideband simulation noise was significantly correlated with that of each type of narrowband simulation noise (0.2 kHz, *r* = 0.7267; 0.4 kHz, *r* = 0.8036; 0.8 kHz, *r* = 0.6407; 1.6 kHz, *r* = 0.5685; 3.2 kHz, *r* = 0.4616), and the correlation coefficient increased as the center frequency of narrowband noise decreased.

Therefore, high-frequency components contribute less to the ability to temporally store wideband features than low-frequency components.

## 5. Discussion

The results in the present study show that when the duration of CIC is long enough, human listeners are easily able to detect the CIC embedded in wideband noise signals even when an ID was introduced. These results provide support to the deduction in Huang et al., (2009) [10] and Li et al., (2013) [11], showing that the duration threshold for detecting a sudden drop in band-limited noise labels becomes gradually larger as the center frequency increases from 0.2 to 3.2 kHz.

Since phase-locking tended to break down as the frequency increased [21,25], the longest IDs for detecting the CIC in high-frequency noise were shorter than those in low-frequency noise. As there was a reduction in the simultaneous correlation coefficient, the loss of phase-locking with the increase in frequency could partially explain the decrease in perceptual responses to the CIC.

The most interesting aspect of this result is that the longest interaural delay for narrowband noise signals was highly correlated with the longest interaural delay for wideband noise signals, and low-frequency components and high-frequency components all contribute to the auditory storage of wideband features. When the longest binaural delay is introduced, the binaural delay of the low-frequency component lasts longer than that of the high-frequency component. When a very short binaural delay is introduced, high-frequency components may not be detected. However, the duration of different frequency components has different benefits for auditory cues. Our results are also consistent with the binary theory of sound localization [34], suggesting that the time difference between sounds is an important cue only at low frequencies. It also supports the results in Wightman et al., (1992) [35] and demonstrates the dominance of low-frequency temporal cues. Since the temporal dynamic of the acoustic signal recognition function was significantly correlated with the listeners’ auditory ability to temporally store acoustic features [11], the relationship between the ability of auditory storage of wideband features and the contribution of each frequency component in the auditory processes of lower-level transient storage of acoustic fine features was important to discriminate what contributes to the sound localization and detection in the auditory scene analysis.

Furthermore, in a daily conversation environment with target stimuli and disruptive stimuli, which are full of high-frequency and low-frequency complex waveforms, the ability to transiently store acoustic features is crucial and functional in noisy conditions. Therefore, the auditory system must be tolerant of disruptive stimuli. Hence, the results of the present study may provide useful insights for further research on speech recognition in noise reverberant environments. The study may also be extended into investigation into the ability of auditory transient storage of acoustic feature interactions in both acoustic modeling studies and neurobiological studies.

## 6. Limitations and Future Work

Notably, some complex auditory signals (e.g., speech) contain both frequency and amplitude modulations. Moreover, the sensitivity to changes in interaural correlation in frequency-gliding sound has not been investigated yet. As shown, the auditory system is capable of integrating binaural information across different frequency channels, thus, we hypothesized that human listeners could hear a dynamic change in interaural correlation in noises with center frequency varying unidirectionally when both the spectral and temporal integrations are involved. However, whether the results and the calculation model will change stably within the same scope require further verification.

Although the results showed non-negligible tendencies, there was still a large part of the variability that could not be well explained. Future research will further refine the influencing factors and expand the number of participants to reduce this variability.

In addition, we only included normal hearing younger adults in the present study. The preliminary findings of the current exploratory study have implications for future studies on aging people, and/or hearing impairment populations. For example, the ability of transient auditory storage of acoustic features for older adults with normal hearing can also be estimated using the proposed “binaural integration paradigm” in the present study, and the computational model and logical paradigm in the current study can be adjusted to account for the influences of aging-related decline in binaural processing. Moreover, this paradigm may be used to explore the clinical implications, individuals with various degrees of hearing loss might be considered to be tested and investigate how the computational model can be modified to account for the hearing loss-related change.

## 7. Conclusions

The present study attempted to advance our understanding of how human listeners are able to temporally recognize rapid changes in interaural configurations. Experiments were conducted to examine whether the auditory ability to transiently store acoustic fine features could be maintained when an interaural delay was introduced.

It was found that a binaural gap CIC between interaural correlated narrowband or wideband noise was detectable even when introducing the longest interaural delay. The results indicate that the ability to transiently store information on acoustic features is frequency-dependent and this persistence of low-frequency features lasts longer than that of high-frequency features. The results of the present study also show that low-frequency information seems to dominate perception and is more distinguishable in length than high-frequency components. Moreover, the longest interaural delay for narrowband noise signals was highly correlated with the longest interaural delay for wideband noise signals in this dynamic process of auditory perception. Furthermore, the present study established a computational model to initialize the relationship between the contribution of low-frequency and high-frequency components to the auditory storage of wideband features. The computational description models will provide a much more intuitive understanding of partial research of auditory processing, which will contribute to improving our understanding of how acoustic features are processed and how the sensitivity of the transient memory of acoustic features is represented.

## Data Availability

The raw data supporting the conclusions of the current study will be made available by the corresponding author on reasonable request, without undue reservation.
